# Association of initial e-cigarette and other tobacco product use with subsequent cigarette smoking in adolescents: a cross-sectional, matched control study

**DOI:** 10.1136/tobaccocontrol-2019-055283

**Published:** 2020-03-17

**Authors:** Lion Shahab, Emma Beard, Jamie Brown

**Affiliations:** 1 Department of Behavioural Science and Health, University College London, London, UK; 2 SPECTRUM Consortium, UK – Shaping Public Health Policies To Reduce Inequalities and Harm, UK

**Keywords:** electronic nicotine delivery devices, harm reduction, priority/special populations, non-cigarette tobacco products

## Abstract

**Introduction:**

This study assessed whether initiating e-cigarette use increases the uptake of cigarette smoking in US adolescents compared with behavioural and synthetic controls.

**Methods:**

Data come from 78 265 adolescents in the National Youth Tobacco Survey (2014–2017) of whom 38 630 provided information about the first tobacco product they had used in 2014/15. Ever, past 30 day and established (30 day use and 100+ lifetime cigarettes) cigarette smoking was compared in adolescents who first used an e-cigarette (exposure group), a non-cigarette combustible (CT) or other non-combustible tobacco (NT) product (behavioural controls), and propensity score matched adolescents without initial e-cigarette use (synthetic controls).

**Results:**

Relative to behavioural controls, adolescents who tried e-cigarettes first were less likely to have ever smoked cigarettes (26% vs CT (42.4%; OR 0.48, 95% CI 0.40 to 0.57), or NT initiators (52.7%; OR 0.32, 95% CI 0.26 to 0.39)), to be past 30 day (6% vs CT (11.9%; OR 0.48, 95% CI 0.36 to 0.62), or NT initiators (20.0%; OR 0.26, 95% CI 0.19 to 0.35)) or be established cigarette smokers (0.7% vs CT (3.9%; OR 0.17, 95% CI 0.10 to 0.30), or NT initiators (8.4%; OR 0.08, 95% CI 0.04 to 0.13)). E-cigarette initiators were also less likely than synthetic controls (without initial e-cigarette use) to have ever smoked cigarettes (OR 0.76, 95% CI 0.62 to 0.93), be past 30 day (OR 0.71, 95% CI 0.55 to 0.91) or be established cigarette smokers (OR 0.26, 95% CI 0.13 to 0.51).

**Conclusion:**

Less than 1% of US adolescents who use e-cigarettes first were established cigarette smokers. They were less likely to be smokers than adolescents who tried other combustible or non-combustible tobacco products first and propensity score matched adolescents without initial e-cigarette use.

## Introduction

There is considerable debate about the impact of e-cigarettes on youth smoking. A contentious point is whether e-cigarettes act as a gateway and increase the likelihood of subsequent cigarette smoking. A large number of studies have shown that e-cigarette experimentation is longitudinally associated with uptake of cigarettes.^1–9^ There are two important limitations of work that has been undertaken to investigate this gateway hypothesis. First, most studies have considered the impact of e-cigarettes on ever use of cigarettes—that is, initiation—but ignore their effect on continued use.^3 10^ This is problematic because it does not allow for a more nuanced analysis of the impact of e-cigarette use on smoking trajectories and health implications, as only regular cigarette use will result in subsequent premature death and disability. Second, a causal association between e-cigarette use and subsequent cigarette smoking cannot be tested directly; it would be unethical (and impractical) to conduct a randomised controlled trial allocating non-smoking adolescents to receive e-cigarettes or not to see whether this leads to uptake of smoking. In the absence of direct tests of the gateway hypothesis, longitudinal data alone are of limited use. Even if e-cigarette use precedes cigarette use, this does not mean that e-cigarettes “caused” subsequent smoking. For example, it could be that adolescents who try e-cigarettes would have tried cigarettes anyway due to common liability such as genetic vulnerability or environmental factors.^11^ While regression analysis can account for some of this confounding, it is still subject to biased estimates in the presence of misclassification and residual confounding,^12^ which undermine confidence in these results claiming to show causal associations.^13^ It is therefore important to triangulate results using different methodologies to account for confounding in order to evaluate the impact that e-cigarettes may have.

The nature of any gateway depends on the counterfactual scenario in which those same adolescents would not have used e-cigarettes. That is, it may be the case that adolescents who would not have used anything at all may be more likely to start smoking cigarettes in the presence of e-cigarettes, consistent with a gateway towards smoking. By contrast, adolescents who would have first used some tobacco product may be less likely to start smoking in the presence of e-cigarettes, consistent with a gateway away from smoking. While opposite effects are also possible (if unlikely), crucially, it is the aggregate effect of both directional pathways that will determine the health impact that e-cigarettes have—that is, do e-cigarettes direct more adolescents to try cigarettes than divert adolescents away from going on to try them, or vice versa?

One way to address this problem is to use matched controls. First, we can match individuals exposed to a putative risk factor with controls not exposed to this risk factor but who are otherwise similar, based on behavioural factors. Here, we suggest comparing adolescents who have initially used e-cigarettes (exposure group) with those who have used another non-combustible tobacco product (behavioural controls) and determine subsequent smoking rates. This will, at least in part, account for the confounding with internal factors such as experimentation (the idea that adolescents who try things will try other things) and environmental factors (increased likelihood of trying products if these are easily available) as both apply to these two groups of adolescents. To account for the great variety of tobacco products and to provide context, we also compared subsequent smoking rates among adolescents who initiated with cigarettes or other combustible tobacco products.

Second, we can match exposed with unexposed individuals using propensity score matching (PSM). PSM is a statistical modelling technique, which uses propensity scores derived from a number of theoretically important characteristics linked with the outcome of interest, that is, cigarette smoking, but not with exposure, that is, e-cigarette use, to identify synthetic controls very similar to the exposed group.^14^ PSM allows a non-randomised observational study to mimic some of the characteristics of a randomised controlled trial. For example, it reduces (but does not eliminate) the risk of confounding and should therefore provide less biased effect estimates than simply following up a self-selecting group of adolescents who have or have not chosen to try an e-cigarette. In contrast to standard multivariable regression analysis, PSM has the advantage that it is less affected by model misspecifications and provides more robust estimates in settings where events are rare relative to the number of confounders, where confounders are widely distributed, and where exposure and confounders are highly correlated,^15–17^ as is the case in this scenario.

Given the limitations of extant research, this study therefore aims to:

1. Compare ever, past 30 day or established cigarette smoking rates of adolescents who first used e-cigarettes with adolescents who first used cigarettes or other combustible or non-combustible tobacco products (behavioural controls).
2. Compare ever, past 30 day or established cigarette smoking rates of adolescents with initial e-cigarette use and matched adolescents with no initial e-cigarette use, selected using PSM (synthetic controls). This analysis was also repeated for initial use of other tobacco products.

## Methods

### Study design and participants

The National Youth Tobacco Survey (NYTS) is an annual, nationally representative, self-administered survey of US middle and high school students aged 9 years and above. Sample selection uses a stratified, three stage cluster design, which proceeds probabilistically without replacement where a primary sampling unit (PSU) is selected within each stratum, a school within each PSU and classes within each school. Participation is voluntary for schools and students (see https://www.cdc.gov/tobacco/data_statistics/surveys/nyts/index.htm for details). This analysis uses data from 2014 to 2017.

### Measures

#### Explanatory variables

In 2014 and 2015 only, adolescents were asked which of a number of tobacco products, if any, they had tried first. This was recoded to produce an exposure variable, dividing adolescents into those who had never used a tobacco product, those who had used an e-cigarette first and those who had used a cigarette first, or other combustible (cigars, cigarillos, little cigars, hookah/waterpipe with tobacco, pipe, bidis) or non-combustible (chewing tobacco, snuff, dip, snus, dissolvable tobacco) tobacco products first.

#### Outcome variables

Adolescents were asked if they had ever tried a cigarette, even a puff or two. Those who said “yes” were classified as ever cigarette smokers. Those who had smoked at least one cigarette in the past 30 days were classified as such, and those who had also smoked >100 cigarettes in their lifetime were classified as established cigarette smokers. As for cigarette smoking, ever and past 30 day use was also assessed for other product use. In 2014 and 2015, adolescents who had indicated ever use of any product but reported they had never tried any product in response to the question about which tobacco product they had used first were marked as inconsistent.

#### Covariates

The following potential confounding variables were assessed across all four waves:

- Age (from 8 years through to 19 years of age or above)
- Sex (male/female)
- Ethnicity (non-Hispanic white, non-Hispanic black, Hispanic, non-Hispanic other)
- Grade (6th grade through to 12th grade)
- School type (middle or high school)
- Future smoking susceptibility measured by three items: likelihood of (a) smoking a cigarette in the next year; (b) trying a cigarette soon; (c) trying a cigarette if offered by a friend (definitely yes, probably yes, probably no, definitely no)
- Environmental exposure to tobacco (living with someone who uses tobacco; yes/no)
- Perceived health effects of smoking, measured by two items: (a) agreement with the statement “All tobacco products are dangerous” (strongly agree, agree, disagree, strongly disagree); and (b) views on harmfulness of breathing smoke from other people’s cigarettes or other tobacco products (no harm, little harm, some harm, a lot of harm).

### Analysis

**Figure 1:**
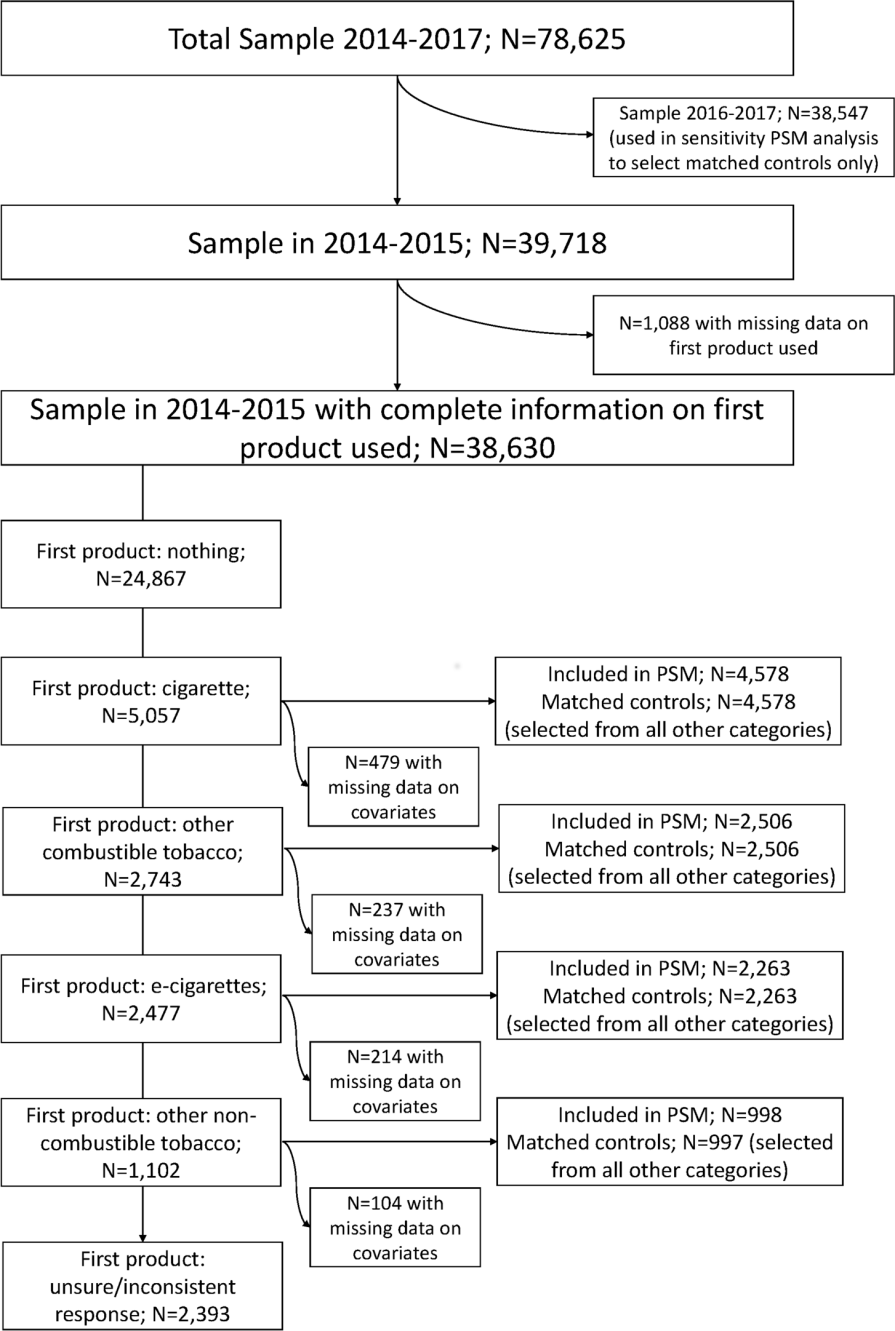
Flow diagram of participants included in main analysis. PSM, propensity score matching.

Descriptive and inferential statistics were computed with the complex samples procedure in SPSS (Statistical Package for the Social Sciences) to account for the sampling design and non-response and were weighted to be representative for the underlying population, using weights, stratum and cluster (PSU) information provided by the NYTS. Sample characteristics were compared by year and, for 2014 and 2015, by first product use with χ^2^ test, or logistic regression in adjusted analyses. PSM was performed as previously described.^18^ Briefly, control groups matched to the indicator groups—(a) adolescents who first used an e-cigarette; (b) a cigarette; (c) other combustible tobacco products; or (d) other non-combustible tobacco products—were selected. This was based on all covariates using PSM via the “psmatching” custom dialogue in SPSS which performs analysis in R through an SPSS R-plugin, following a standard method,^19^ applied to the 2014 and 2015 data. Figure 1 provides a flow diagram of participants included in this analysis.

Model adequacy checks were performed to ascertain that appropriate balance of covariates was achieved via the matching procedure. Five diagnostic plots were inspected: (a) propensity scores histograms in both groups before and after matching; (b) individual propensity score dotplots of individuals in matched and unmatched control and indicator groups; (c) histograms of standardised differences of all terms (covariates, quadratic terms and interactions) before and after matching; (d) a dotplot of the magnitude of standardised differences before and after matching for each covariate; and (e) a lineplot of standardised mean difference before and after matching. In addition, the relative multivariate imbalance L1 measure (with lower values indicating better matching) and χ^2^ balance test (with non-significant values indicating good variable balance) were computed.

Once PSM was completed, ever, past 30 day or established cigarette smoking rates were compared in adolescents who had used an e-cigarette first with matched control adolescents who had not used an e-cigarette first, and in adolescents who had used other tobacco products first with respective matched control adolescents who had not used other tobacco products first. Odds ratios were computed using logistic regression. For e-cigarette propensity score matching results only, Bayes factors (BF) were calculated to determine whether data were supportive of the null (BF <1/3) or alternative hypothesis (BF >3), or were insensitive (BF >1/3 and <3), using an online calculator (http://www.lifesci.sussex.ac.uk/home/Zoltan_Dienes/inference/Bayes.htm) and following standard methodology.^20^ We used a conservative approach with half-normal distribution, with the mode at 0 (no effect), and the standard deviation equal to the alternative hypothesis (OR 3.5 and 4.28 for ever and past 30 day cigarette use, based on a previous meta-analysis).^10^ We also calculated the range of expected effect sizes, which would be insensitive to distinguish between the null and alternative hypothesis to provide a robustness region of the likely effect.

We conducted two planned sensitivity analyses. First, the comparison of smoking rates among exposed and matched control groups derived from propensity score analysis was repeated, including additional adjustment for specified covariates.^21^ Second, the PSM analysis was repeated, also including adolescents from 2016 and 2017, to provide up-to-date results. This analysis was less definitive because the question assessing which tobacco use came first was removed in the latter two waves. The matched controls were selected from adolescents between 2014 through to 2017 who did not first use an e-cigarette or may not have first used an e-cigarette, using the method outlined above.

All analyses were performed using SPSS version 21 and R version 2.14.2, missing data removed listwise and family-wise error rate corrected using the false discovery rate.^22^

### Study registration

The analysis plan for this study was pre-specified and logged at the Open Science Framework (https://osf.io/9zsw3).

## Results

Sociodemographic characteristics of the NYTS sample were stable between 2014–2017 (table 1). With the exception of 2015, there was a general trend of a decrease in the ever or past 30 day use of any product, in particular cigarettes and other combustible tobacco use, with no changes for non-combustible tobacco and increased e-cigarette use from 2014 onwards (table 1).

**Table 1:** Sociodemographic and tobacco use characteristics by survey year*

	Total (n=78 265)	2014 (n=22 007)	2015 (n=17 711)	2016 (n=20 625)	2017 (n=17 872)	P value
	% (95% CI)					
**Sociodemographic characteristics**						
Sex						0.71
Female	49.2 (48.4 to 49.9)	49.8 (48.0 to 51.5)	48.8 (47.2 to 50.4)	49.4 (48.2 to 50.5)	48.7 (47.4 to 49.9)	
Male	50.8 (50.1 to 51.6)	50.2 (48.5 to 52.0)	51.2 (52.8 to 49.6)	50.6 (49.5 to 51.8)	51.3 (50.1 to 52.6)	
Age (years)						0.931
≤12	19.2 (17.9 to 20.5)	18.7 (16.4 to 21.2)	18.6 (15.7 to 21.8)	19.3 (16.8 to 22.0)	20.1 (17.7 to 22.9)	
13	15.0 (13.9 to 16.1)	15.3 (12.9 to 18.0)	14.8 (12.7 to 17.2)	15.2 (13.2 to 17.5)	14.7 (13.0 to 16.6)	
14	15.0 (14.4 to 15.5)	14.7 (13.5 to 16.0)	15.5 (14.3 to 16.9)	15.1 (14.0 to 16.1)	14.6 (13.6 to 15.6)	
15	15.1 (14.3 to 15.9)	14.9 (13.4 to 16.5)	15.1 (13.5 to 16.9)	14.8 (13.3 to 16.4)	15.6 (14.1 to 17.2)	
16	14.1 (13.4 to 14.9)	14.1 (12.6 to 15.8)	13.7 (12.2 to 15.5)	14.5 (13.2 to 16.0)	14.1 (12.7 to 15.6)	
17+	21.7 (20.5 to 22.9)	22.3 (19.9 to 25.0)	22.2 (19.4 to 25.4)	21.1 (19.1 to 23.4)	20.9 (18.8 to 23.2)	
Ethnicity						0.953
Non-Hispanic white	57.0 (54.7 to 59.4)	58.3 (52.5 to 63.8)	56.8 (50.7 to 62.7)	56.1 (50.3 to 61.7)	57.0 (49.8 to 63.8)	
Non-Hispanic black	13.9 (12.5 to 15.4)	15.3 (12.0 to 19.4)	14.2 (10.5 to 18.9)	13.0 (10.0 to 16.9)	13.1 (10.4 to 16.3)	
Hispanic	23.9 (22.2 to 25.6)	21.9 (18.4 to 25.8)	23.8 (19.3 to 29.0)	25.4 (21.1 to 30.3)	24.3 (19.1 to 30.4)	
Non-Hispanic other	5.2 (4.6 to 5.9)	4.5 (3.5 to 5.8)	5.2 (3.9 to 6.9)	5.4 (4.4 to 6.7)	5.7 (4.3 to 7.3)	
School†						0.999
Middle school (grade 6–8)	44.1 (41.2 to 47.0)	43.9 (38.1 to 49.9)	44.1 (37.6 to 50.8)	44.2 (39.0 to 49.6)	44.1 (38.8 to 49.4)	
High school (grade 9–12)	55.9 (53.0 to 58.8)	56.1 (50.1 to 61.9)	55.9 (49.2 to 62.4)	55.8 (50.4 to 61.0)	55.9 (50.6 to 61.2)	
**Tobacco use characteristics**						
Ever use						
Any product	34.1 (32.9 to 35.3)	34.9 (33.0 to 37.0)	37.0 (34.2 to 39.8)	33.7 (31.7 to 35.7)	30.7 (28.5 to 33.0)	0.003
Cigarettes	20.3 (19.3 to 21.3)	22.4 (20.8 to 24.2)	22.0 (19.8 to 24.4)	20.0 (18.0 to 22.2)	16.8 (15.0 to 18.7)	0.001
Other combustible‡	21.1 (20.2 to 22.0)	23.5 (22.0 to 25.1)	22.8 (20.7 to 25.1)	20.6 (19.1 to 22.2)	17.5 (15.9 to 19.1)	<0.001
E-cigarettes	22.9 (21.9 to 24.0)	19.9 (18.1 to 21.8)	27.1 (24.9 to 29.5)	23.1 (21.4 to 24.8)	21.5 (19.5 to 23.7)	<0.001
Other non-combustible§	9.4 (8.7 to 10.2)	9.6 (8.3 to 11.1)	9.2 (7.7 to 11.0)	9.7 (8.3 to 11.4)	9.1 (7.5 to 11.1)	0.952
Past 30 day use						
Any product	15.9 (15.1 to 16.7)	17.3 (16.0 to 18.6)	17.5 (15.7 to 19.4)	14.9 (13.6 to 16.3)	13.9 (12.4 to 15.5)	0.002
Cigarettes	5.8 (5.4 to 6.3)	6.3 (5.6 to 7.0)	6.2 (5.2 to 7.5)	5.5 (4.6 to 6.4)	5.3 (4.5 to 6.1)	0.288
Other combustible‡	8.6 (8.2 to 9.1)	10.0 (9.2 to 10.9)	8.9 (8.0 to 10.0)	8.4 (7.6 to 9.3)	7.2 (6.4 to 8.2)	<0.001
E-cigarettes	9.2 (8.6 to 9.9)	9.3 (8.0 to 10.8)	11.3 (10.1 to 12.7)	8.2 (7.4 to 9.2)	8.1 (6.8 to 9.5)	0.001
Other non-combustible§	4.2 (3.8 to 4.6)	4.4 (3.7 to 5.2)	4.2 (3.3 to 5.4)	4.3 (3.5 to 5.2)	3.9 (3.2 to 4.9)	0.908
First tried¶				–	–	
Nothing	64.1 (62.4 to 65.7)	64.8 (62.8 to 66.8)	63.3 (60.4 to 66.1)			0.387
Cigarettes	13.1 (12.2 to 14.0)	13.9 (12.7 to 15.1)	12.3 (11.0 to 13.7)			0.107
Other combustible‡	7.3 (6.7 to 8.0)	6.6 (5.8 to 7.4)	8.0 (7.0 to 9.1)			0.031
E-cigarettes	6.6 (6.0 to 7.2)	5.6 (4.9 to 6.4)	7.6 (6.8 to 8.4)			0.001
Other non-combustible§	3.1 (2.6 to 3.8)	3.3 (2.5 to 4.2)	3.0 (2.2 to 4.1)			0.709
Unsure/inconsistent**	5.9 (5.4 to 6.4)	5.9 (5.3 to 6.6)	5.8 (5.1 to 6.7)			0.945

*Weighted percentages, raw N.

†Excludes unspecified grades (N=77).

‡Includes cigars, cigarillos, little cigars, hookah/waterpipe with tobacco, pipe, bidis.

§Includes chewing tobacco, snuff, dip, snus, dissolvable tobacco.

¶Only asked in 2014/2015 (total n=38 630).

**Those who could not remember (n=590) or who had indicated any use of a tobacco product but claimed they had never tried a product first (n=1803).

The most common initiation products were cigarettes, followed by other combustibles, e-cigarettes and other non-combustible tobacco (table 1). From 2014 to 2015, initiation with other combustible tobacco and e-cigarettes increased significantly, with other product categories remaining constant. Male adolescents were more likely than female adolescents to have used other non-combustible tobacco first, and vice versa for cigarettes, but overall female students were less likely than male students to have initiated any product use (table 2). Initiation with any product increased with increasing age and was higher in high than middle school. Non-Hispanic black adolescents were least likely to have initiated product use with cigarettes but most likely to have used other combustible tobacco first. E-cigarettes were most likely to be used first by Hispanic adolescents and other non-combustible tobacco by non-Hispanic white adolescents. Overall, adolescents with a non-Hispanic other background were least likely to have used any product first.

**Table 2:** Prevalence of first product tried by sociodemographic characteristics in 2014 and 2015†

Sociodemographic characteristics	Nothing (n=24 867)	Cigarette (n=5057)	Other combustible tobacco‡ (N=2734)	E-cigarettes (n=2477)	Other non-combustible tobacco§ (n=1102)	Unsure/inconsistent (2393)
	% (95% CI)					
Sex	***	***			***	***
Female	66.4 (64.7 to 68.0)	14.0 (13.0 to 15.1)	7.1 (6.4 to 7.8)	6.3 (5.7 to 7.0)	1.0 (0.8 to 1.3)	5.2 (4.7 to 5.8)
Male	61.9 (59.9 to 63.9)	12.2 (11.3 to 13.1)	7.5 (6.8 to 8.3)	6.8 (6.1 to 7.5)	5.2 (4.3 to 6.4)	6.4 (5.8 to 7.1)
Age (years)	***	***	***	***	***	***
≤12	87.5 (85.9 to 88.9)	4.4 (3.8 to 5.2)	1.4 (1.1 to 1.8)	2.9 (2.4 to 3.5)	1.3 (0.9 to 1.8)	2.5 (2.1 to 3.0)
13	78.0 (75.5 to 80.4)	7.0 (6.0 to 8.1)	3.4 (2.7 to 4.2)	5.4 (4.5 to 6.5)	1.7 (1.1 to 2.6)	4.5 (3.7 to 5.4)
14	70.7 (68.7 to 72.6)	9.8 (8.6 to 11.1)	4.7 (4.0 to 5.5)	7.0 (6.0 to 8.1)	1.9 (1.4 to 2.7)	5.9 (5.0 to 6.9)
15	59.3 (56.6 to 62.0)	14.7 (13.2 to 16.4)	7.8 (6.6 to 9.1)	8.5 (7.3 to 9.7)	2.9 (2.1 to 3.8)	6.8 (6.0 to 7.8)
16	50.3 (48.2 to 52.3)	19.3 (17.5 to 21.2)	9.9 (8.6 to 11.4)	8.9 (7.9 to 10.1)	4.6 (3.5 to 6.0)	7.0 (6.2 to 7.9)
17+	42.6 (40.4 to 44.8)	21.7 (20.1 to 23.4)	14.6 (13.4 to 15.9)	7.2 (6.3 to 8.3)	5.7 (4.7 to 7.0)	8.1 (7.1 to 9.1)
Ethnicity	***	*	***	***	***	***
Non-Hispanic white	65.5 63.3 to 67.6)	13.5 (12.4 to 14.7)	6.5 (5.7 to 7.3)	6.6 (5.9 to 7.4)	4.5 (3.7 to 5.5)	3.4 (3.0 to 3.8)
Non-Hispanic black	62.2 (59.0 to 65.3)	11.1 (9.9 to 12.4)	10.6 (9.2 to 12.2)	4.5 (3.8 to 5.4)	0.7 (0.5 to 1.0)	11.0 (9.6 to 12.5)
Hispanic	59.6 (57.6 to 61.6)	14.0 (12.9 to 15.3)	8.2 (7.2 to 9.3)	8.2 (7.4 to 9.1)	1.6 (1.3 to 2.0)	8.3 (7.6 to 9.2)
Non-Hispanic other	70.2 (63.7 to 76.0)	11.5 (8.8 to 14.9)	4.3 (3.4 to 5.4)	5.3 (3.9 to 7.3)	1.4 (0.9 to 2.3)	7.3 (5.4 to 9.8)
School	***	***	***	***	***	***
Middle school (grade 6–8)	80.4 (78.5 to 82.2)	6.7 (5.9 to 7.7)	2.7 (2.3 to 3.1)	4.6 (4.1 to 5.2)	1.6 (1.2 to 2.2)	4.0 (3.5 to 4.5)
High school (grade 9–12)	51.3 (49.5 to 53.1)	18.1 (16.8 to 19.4)	10.9 (10.1 to 11.7)	8.1 (7.3 to 8.9)	4.4 (3.6 to 5.4)	7.3 (6.6 to 8.0)

*p<0.05; **p<0.01; ***p<0.001 for within group (product use) analyses.

†Weighted percentages, raw N, missing data (n=1088).

‡Includes cigars, cigarillos, little cigars, hookah/waterpipe with tobacco, pipe, bidis.

§Includes chewing tobacco, snuff, dip, snus, dissolvable tobacco.

### Comparison of smoking rates by first product used (behavioural control)

Compared with behavioural controls, adolescents who initiated with e-cigarettes were less likely to have ever smoked cigarettes than those who first used non-cigarette combustible tobacco (OR 0.48, 95% CI 0.40 to 0.57), other non-combustible tobacco (OR 0.32, 95% CI 0.26 to 0.39), those who were unsure or had provided an inconsistent response (OR 0.50, 95% CI 0.42 to 0.60) and, by definition, those who had used cigarettes first (table 3). Similarly, compared with all other groups, with the exception of those who were unsure or had provided an inconsistent response, adolescents who had used an e-cigarette first were also less likely to be past 30 day or established cigarette smokers compared with those who had first used a cigarette (OR 0.15, 95% CI 0.12 to 0.18, and OR 0.04, 95% CI 0.03 to 0.07, respectively), other combustible tobacco (OR 0.48, 95% CI 0.36 to 0.62, and OR 0.17, 95% CI 0.10 to 0.30, respectively) or other non-combustible tobacco (OR 0.26, 95% CI 0.19 to 0.3, and OR 0.08, 95% CI 0.04 to 0.13, respectively). E-cigarette initiators were also less likely to be established cigarette smokers (but not past 30 day smokers) than those who were unsure or who had provided an inconsistent response (OR 0.46, 95% CI 0.25 to 0.87) (table 3).

**Table 3:** Prevalence of cigarette and e-cigarette use and smoking susceptibility characteristics by first product tried in 2014 and 2015*

	Nothing (n=24 867)	Cigarette (n=5057)	Other combustible tobacco† (n=2734)	E-cigarettes (n=2477)	Other non-combustible tobacco‡ (n=1102)	Unsure/inconsistent (2393)	P value
	% (95% CI)						
**Cigarette smoking**							
Ever use	–	a	b	c	d	b	<0.001
		100	42.4 (39.5 to 45.4)	26.0 (23.4 to 28.7)	52.7 (48.4 to 57.0)	41.2 (37.9 to 44.6)	
Past 30 day use	–	a	b	c	d	c	<0.001
		30.6 (28.5 to 32.8)	11.9 (10.1 to 14.0)	6.0 (4.9 to 7.4)	20.0 (16.5 to 24.2)	5.2 (4.2 to 6.4)	
Established past 30 day use	–	a	b	c	d	d	<0.001
		14.3 (12.7 to 16.0)	3.9 (3.0 to 4.9)	0.7 (0.4 to 1.1)	8.4 (6.5 to 10.8)	1.5 (1.0 to 2.2)	
**E-cigarette use**							
Ever use	–	a	b	c	b	d	<0.001
		65.2 (62.2 to 68.1)	54.1 (49.6 to 58.6)	100	53.7 (48.1 to 59.2)	42.6 (38.6 to 46.6)	
Past 30 day use	–	a	b	c	b	d	<0.001
		31.7 (28.8 to 34.8)	24.1 (21.6 to 26.7)	38.9 (35.6 to 42.3)	26.8 (22.4 to 31.8)	12.2 (10.5 to 14.2)	
**Smoking susceptibility characteristics**							
Any tobacco use at home	a	b	c, d	c	b	d	<0.001
Yes	33.6 (32.2 to 35.1)	65.0 (62.8 to 67.1)	54.7 (52.1 to 57.3)	55.9 (53.2 to 58.7)	66.6 (62.3 to 70.6)	50.2 (47.4 to 53.0)	
No	66.4 (64.9 to 67.8)	35.0 (32.9 to 37.2)	45.3 (42.7 to 47.9)	44.1 (41.3 to 46.8)	33.4 (29.4 to 37.7)	49.8 (47.0 to 52.6)	
Smoke cigarette next year	a	b	c	d	e	f	<0.001
Definitely yes	0.1 (0.0 to 0.1)	16.8 (15.1 to 18.5)	5.1 (4.1 to 6.4)	2.6 (2.0 to 3.4)	11.4 (8.9 to 14.5)	2.5 (1.8 to 3.4)	
Probably yes	0.7 (0.6 to 0.8)	20.4 (18.8 to 22.0)	12.4 (10.9 to 14.1)	8.9 (7.5 to 10.5)	15.4 (12.9 to 18.3)	5.7 (4.7 to 6.9)	
Probably not	11.4 (10.7 to 12.2)	27.3 (25.4 to 29.3)	29.3 (26.9 to 31.8)	34.5 (31.9 to 37.2)	31.7 (28.1 to 35.6)	26.9 (24.5 to 29.5)	
Definitely not	87.8 (87.1 to 88.6)	35.5 (33.4 to 37.7)	53.1 (50.3 to 56.0)	54.0 (50.7 to 57.2)	41.5 (37.8 to 45.3)	64.9 (62.0 to 67.7)	
Will try cigarette soon	a	b	c	d	e	f	<0.001
Definitely yes	0.1 (0.0 to 0.1)	16.0 (14.5 to 17.6)	4.9 (3.9 to 6.1)	2.3 (1.7 to 3.1)	8.8 (6.9 to 11.1)	2.5 (1.9 to 3.4)	
Probably yes	1.2 (1.1 to 1.4)	16.7 (15.2 to 18.3)	10.2 (8.9 to 11.7)	9.1 (7.6 to 10.8)	15.7 (12.9 to 19.1)	7.1 (5.8 to 8.7)	
Probably not	12.9 (12.2 to 13.6)	31.2 (29.5 to 32.9)	30.5 (28.0 to 33.1)	35.9 (33.3 to 38.6)	31.6 (27.8 to 35.6)	26.5 (24.3 to (28.8)	
Definitely not	85.8 (85.0 to 86.6)	36.1 (34.0 to 38.2)	54.4 (51.6 to 57.1)	52.7 (49.5 to 55.8)	43.9 (40.2 to 47.7)	63.8 (61.1 to 66.4)	
If best friend offered a cigarette would smoke it	*a	b	c	d	e	f	<0.001
Definitely yes	0.2 (0.1 to 0.3)	18.2 (16.6 to 20.2)	5.7 (4.6 to 6.9)	3.0 (2.3 to 3.8)	11.9 (9.7 to 14.5)	3.1 (2.4 to 4.1)	
Probably yes	1.3 (1.2 to 1.5)	23.3 (21.6 to 25.2)	15.8 (13.9 to 17.9)	13.0 (11.3 to 14.8)	18.4 (16.0 to 21.1)	9.3 (7.7 to 11.2)	
Probably not	14.6 (13.9 to 15.3)	28.9 (27.3 to 30.6)	28.3 (26.4 to 30.4)	35.5 (32.7 to 38.5)	30.8 (26.6 to 35.2)	28.2 (26.0 to 30.5)	
Definitely not	83.9 (83.1 to 84.6)	29.6 (27.5 to 31.7)	50.2 (47.5 to 52.8)	48.5 (45.3 to 51.8)	39.0 (35.2 to 42.9)	59.4 (56.8 to 61.9)	
All tobacco products are dangerous	a	b	c	d	e	f	<0.001
Strongly agree	68.0 (66.7 to 69.2)	35.2 (33.1 to 37.4)	28.9 (26.8 to 31.1)	30.6 (28.0 to 33.3)	22.4 (19.3 to 25.9)	42.2 (39.7 to 44.8)	
Agree	25.4 (24.3 to 26.4)	43.8 (41.9 to 45.8)	48.7 (46.4 to 51.1)	50.7 (48.3 to 53.1)	48.8 (45.2 to 52.4)	37.6 (34.8 to 40.5)	
Disagree	3.2 (2.9 to 3.6)	15.7 (14.1 to 17.4)	17.2 (15.1 to 19.6)	16.1 (14.2 to 18.2)	21.7 (18.6 to 25.0)	11.4 (9.9 to 13.1)	
Strongly disagree	3.4 (3.0 to 3.9)	5.3 (4.5 to 6.2)	5.2 (4.3 to 6.2)	2.6 (2.0 to 3.5)	7.2 (5.5 to 9.2)	8.8 (7.0 to 10.9)	
Harm caused by breathing smoke	a	b	c	b, c	b, c	d	<0.001
No harm	2.8 (2.5 to 3.2)	6.4 (5.6 to 7.4)	4.9 (4.1 to 5.9)	4.3 (3.1 to 5.9)	7.6 (5.8 to 9.9)	10.9 (9.3 to 12.7)	
Little harm	15.9 (15.0 to 16.8)	22.2 (20.8 to 23.7)	19.4 (17.4 to 21.5)	23.0 (20.9 to 25.3)	23.0 (16.5 to 26.9)	17.2 (15.1 to 19.6)	
Some harm	37.6 (36.6 to 38.6)	37.1 (35.2 to 39.0)	44.1 (41.7 to 46.5)	40.3 (37.8 to 42.8)	40.6 (37.2 to 44.0)	33.5 (30.6 to 36.7)	
A lot of harm	43.7 (42.4 to 45.0)	34.2 (32.4 (36.2)	31.7 (29.3 to 34.2)	32.4 (29.8 to 35.1)	28.9 (25.6 to 32.3)	38.3 (35.7 to 41.0)	

a, b, c, d, e, f, Non-shared letters indicate significant differences (p<0.05) for between group (product use) analyses.

*Weighted percentages, raw N, missing data (n=1088).

†Includes cigars, cigarillos, little cigars, hookah/waterpipe with tobacco, pipe, bidis.

‡Includes chewing tobacco, snuff, dip, snus, dissolvable tobacco.

An unplanned analysis to evaluate reverse effects showed that cigarette initiators were more likely to have ever, or in the past 30 days, used an e-cigarette than all other categories, except e-cigarette initiators (table 3). While they were less likely to have used an e-cigarette in the past 30 days than e-cigarette initiators (OR 0.73, 95% CI 0.62 to 0.86), this effect was less pronounced than the reverse association of initial e-cigarette use with past 30 day cigarette smoking reported above.

### Comparison of smoking rates of adolescents who used e-cigarettes or other tobacco products first and matched adolescents who did not (synthetic control)

PSM was conducted using 2014 and 2015 data, based on all sociodemographic variables reported in table 1 and smoking susceptibility characteristics reported in table 3. As shown, there were clear differences in susceptibility characteristics as a function of the first product used, with those who had not used any products showing lower susceptibility across all measures (table 3). While there were significant differences between other groups, it is noteworthy that the majority of those who had initiated with other combustible tobacco, e-cigarettes and those who were unsure or who had provided an inconsistent response, believed they would definitely not smoke a cigarette soon, next year, or if a best friend offered it. This was not the case for those who initiated with cigarettes or other non-combustible tobacco (table 3).

**Figure 2:**
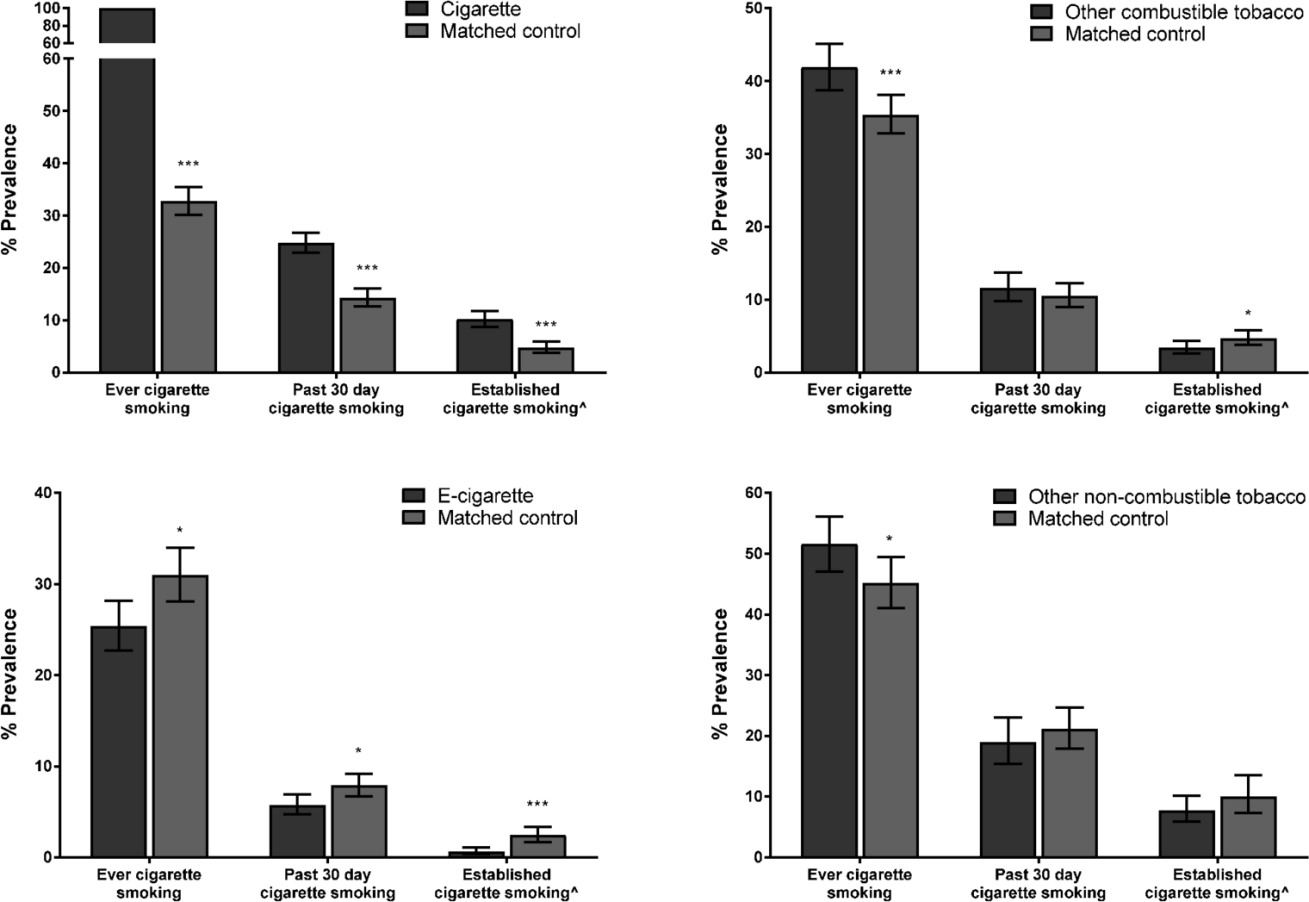
Prevalence of cigarette use by first product tried and propensity scorematched controls selected from 2014 and 2015. Error bars are 95% CI; ***p<0.001; *p<0.05; ˆSmoking in past 30 days and at least 100 cigarettes in lifetime.

After PSM, the overall χ^2^ balance test was non-significant for all groups (p=0.356–0.993), and the L1 measure had reduced in all cases, indicating that covariate imbalance had improved in matched cases, producing a similar propensity score profile in matched adolescents to those who had used a product first (see online supplementary figures S1–S4). Those who had used a cigarette first were more likely to have ever smoked a cigarette (by definition), to be past 30 day (OR 1.97, 95% CI 1.72 to 2.27) or established cigarette smokers (OR 2.27, 95% CI 1.74 to 2.95) than matched controls who had not initiated with cigarettes (figure 2). Similarly, those who had first tried other combustible or non-combustible tobacco were more likely than matched controls to have ever smoked a cigarette (OR 1.32, 95% CI 1.13 to 1.58, and OR 1.29, 95% CI 1.03 to 1.62), with no difference in past 30 day smoking. Those who had initiated with other combustible (but not non-combustible) tobacco products were, however, less likely to be established cigarette smokers than matched controls (OR 0.71, 95% CI 0.51 to 0.98). Those who had initiated with e-cigarettes were consistently less likely than matched controls to have ever smoked a cigarette (OR 0.76, 95% CI 0.62 to 0.93), be a past 30 day (OR 0.71, 95% CI 0.55 to 0.91) or established cigarette smoker (OR 0.26, 95% CI 0.13 to 0.51). Bayesian analysis suggested that, based on data in the literature about the increased risk of cigarette smoking following e-cigarette initiation, the current analysis provides substantial evidence for the null hypothesis (ie, that there is no gateway of the strength postulated for ever (BF=0.01) and past 30 day cigarette smoking (BF=0.03) use). In fact, the range of likely estimates was OR 0.05 to 1.05 for ever and OR 0.07 to 1.09 for past 30 day use. Results were not materially changed when additionally adjusting logistic regression analyses for covariates used in PSM. Differences between e-cigarette initiators and synthetic (matched) controls were very similar when conducting PSM including matched controls from all four waves (see online supplementary figure S5), indicating a consistent association across the whole time period considered.

10.1136/tobaccocontrol-2019-055283.supp1 Supplementary data

## Discussion

The NYTS showed a continuing decrease in both cigarette smoking prevalence and in the use of any tobacco product, despite a concurrent increase in e-cigarette use between 2014 to 2017. This suggests that any gateway effect of e-cigarettes, if present, must be small. Further, despite e-cigarettes being more commonly used than any other product from 2015 onwards, cigarettes remained the most prevalent initiation product in 2014 and 2015, followed by other combustibles.

In line with previous longitudinal analyses, the current cross-sectional matched control study estimated that around a quarter of e-cigarette initiators go on to try cigarettes subsequently.^10^ However, <1% of adolescents trying an e-cigarette first became established cigarette smokers, significantly fewer than in any other product category. The conversion rate from ever to established cigarette smoking was much lower for e-cigarette initiators (2.7%) than for those who tried non-cigarette combustible (9%) or non-combustible tobacco products first (15.9%), indicative of a possible protective effect of e-cigarettes compared with these behavioural controls. This interpretation was supported by PSM analysis, indicating that e-cigarette initiators compared with matched synthetic controls were less likely to try cigarettes or become past 30 day or established cigarette smokers. The same pattern was not observed for initiators with other tobacco products, which appeared to increase the risk of ever-smoking cigarettes compared with matched synthetic controls. In agreement with previous work,^23^ we also found that the association of subsequent use of e-cigarettes was stronger for adolescents initiating with cigarettes than the association of subsequent cigarette smoking for e-cigarette initiators. This underlines the fact that cigarettes act as a much more important gateway for any product use.

The current analysis suggests that the association of e-cigarette initiation with subsequent smoking is largely explained by shared vulnerability such that those who try an e-cigarette first would have gone on to smoke cigarettes anyway. The finding that smoking rates in the synthetic matched control group were higher than in adolescents who tried an e-cigarette first is consistent with the interpretation that the aggregate effect of e-cigarettes is one that leads more adolescents away (for those who would have smoked at any rate) than towards subsequent cigarette smoking (for those who would never have smoked). Our results explain the seemingly opposing observations that e-cigarette use is associated longitudinally with a greater likelihood of starting to smoke cigarettes and that youth cigarette smoking rates have continued to fall over the last decade in countries which have seen an increase in e-cigarette use by adolescents, both in the USA^24^ and elsewhere.^25^ However, the direction of the aggregate effect is likely to depend on the underlying population of e-cigarette initiators. As the proportion of e-cigarette initiators who would never have smoked cigarettes increases, the net effect may swing in the opposite direction, as may be the case following the recent rise in the popularity in so called “mod pods” such as JUUL.^26 27^

Our cross-sectional PSM analysis did not replicate longitudinal analyses, using logistic regression to adjust for confounding.^1 28^ While both approaches are vulnerable to residual confounding and misclassification, which can produce spurious associations,^29^ PSM is more robust than regression analysis in situations with high correlations between exposure and confounders^30^ with a low event rate per confounder,^31^ as is the case for starting to smoke following initial e-cigarette use. Our findings are also at odds with recent analyses of the Population Assessment of Tobacco and Health (PATH) data, which showed that adolescents who initiate e-cigarette use are as likely as those who initiate with other tobacco products to go on to smoke cigarettes.^4 23^ Methodological differences may explain this. Specifically, the PATH analyses excluded users of any tobacco products or cigarettes at baseline, resulting in a self-selected, and thus non-representative, sample of adolescents less vulnerable to tobacco use. The likely effect is a depression in the subsequent uptake of cigarette smoking across all groups (the estimated rate of ever cigarette smoking among e-cigarette initiators of 13.8% and 19.1% in these PATH analyses is lower than comparative figures in the literature of >23%^10^) which may obscure any true effect associated with different product use in the total population of adolescents. There may be threshold effects where adolescents with greater smoking susceptibility will be less or more likely to go on to smoke cigarettes than those with lower smoking susceptibility, depending on the first product used. This is illustrated by the PATH analysis where low susceptibility adolescents initiating with e-cigarettes were more likely than low susceptibility initiators with other products to smoke subsequently, whereas the opposite association was observed for high susceptibility adolescents.^23^ This also highlights the limitations of individual-level analyses in previous and our work; namely, it is likely that the net effect that e-cigarettes have on subsequent smoking rates is a function of the underlying population of adolescents and their susceptibility profile. That is, if e-cigarette initiation occurs mainly among adolescents with a high susceptibility for smoking, the net effect may be one where more adolescents are led away from becoming established smokers than into smoking (gateway out), as is observed in our analysis. However, an opposite net effect into smoking (gateway in) may be observed if more adolescents with a low than high susceptibility for smoking initiate with e-cigarettes. The best way to estimate any true net gateway effect would therefore be to look at population-level associations, for example, with time-series analyses, which avoid individual-level confounding.

This study has limitations. First, although we used synthetic in addition to behavioural controls to address the issue of confounding, PSM is ideally conducting across at least three measurement points to separate exposure, outcome and covariate assessment. In the current analysis, all were measured cross-sectionally, which may have increased the selection of smokers into propensity score matched controls. Second, the primary exposure variable, first product used, may be subject to recall bias. However, both issues would equally apply to the other product categories and could not explain differential associations observed with these products compared with e-cigarettes. Third, while a wide range of covariates where included to determine susceptibility to smoking, not all factors relevant for smoking initiation such as conduct and mental health problems were available.^32^ Lastly, no details on the specific e-cigarettes used were available. Given the wide variety of products with different usage and psychopharmacological profiles—notably the analysis preceded the increase in the popularity of ‘mod pods’ like JUUL—different effects may emerge which would be consistent with a gateway towards cigarette smoking.

In conclusion, this matched control analysis of NYTS data from 2014 to 2017 suggests that for adolescents initiation with e-cigarettes is associated with a reduced risk of subsequent cigarette smoking compared with initiators with other combustible and non-combustible tobacco products use, and propensity score matched adolescents without initial e-cigarette use. This suggests that, over the time period considered, e-cigarettes were unlikely to have acted as an important gateway towards cigarette smoking and may, in fact, have acted as a gateway away from smoking for vulnerable adolescents; this is consistent with the decrease in youth cigarette smoking prevalence over the same time period that youth e-cigarette use increased between 2014 and 2017.

**What this paper adds**

- The debate about the impact of e-cigarettes on youth smoking, in particular gateway effects, is ongoing. Research shows a consistent association of e-cigarette use with subsequent smoking among adolescents.
- Given the limitation of standard observational analyses to address this issue due to confounding, novel analytical techniques are required to assess likely gateway effects in the context of increasing e-cigarette use among adolescents.
- This cross-sectional, matched control study uses both behavioural controls and propensity score matched synthetic controls to show that the postulated gateway effect is likely to be small and that the observed association of e-cigarette use with subsequent smoking largely reflects confounding due to common liability.
