# Supplementary material for: Association of initial e-cigarette and other tobacco product use with subsequent cigarette smoking in adolescents: a cross-sectional, matched control study
Source: Tob Control. 2020 Mar 17;30(2):212–20. doi: 10.1136/tobaccocontrol-2019-055283 (PMC7907552; doi:10.1136/tobaccocontrol-2019-055283)
Supplement: Supplementary data [file tobaccocontrol-2019-055283supp001.pdf]

Supplementary Figures

Figure S1 (a) Distribution of propensity scores of adolescents in 2014/2015 who used cigarettes first (treated) and those who did not (control) before and after matching overlaid kernel density estimate; (b) dotplot of adolescents in either matched or unmatched groups; (c) dotplot of standardized mean differences (Cohen's *d*) for all covariates before and after matching; (d) historgrams with overlaid kernel density estiamtes of standardardised differences before and after matching; (e) lineplot of standardardised differences before and after matching (bold: standardized differences that increase after matching)

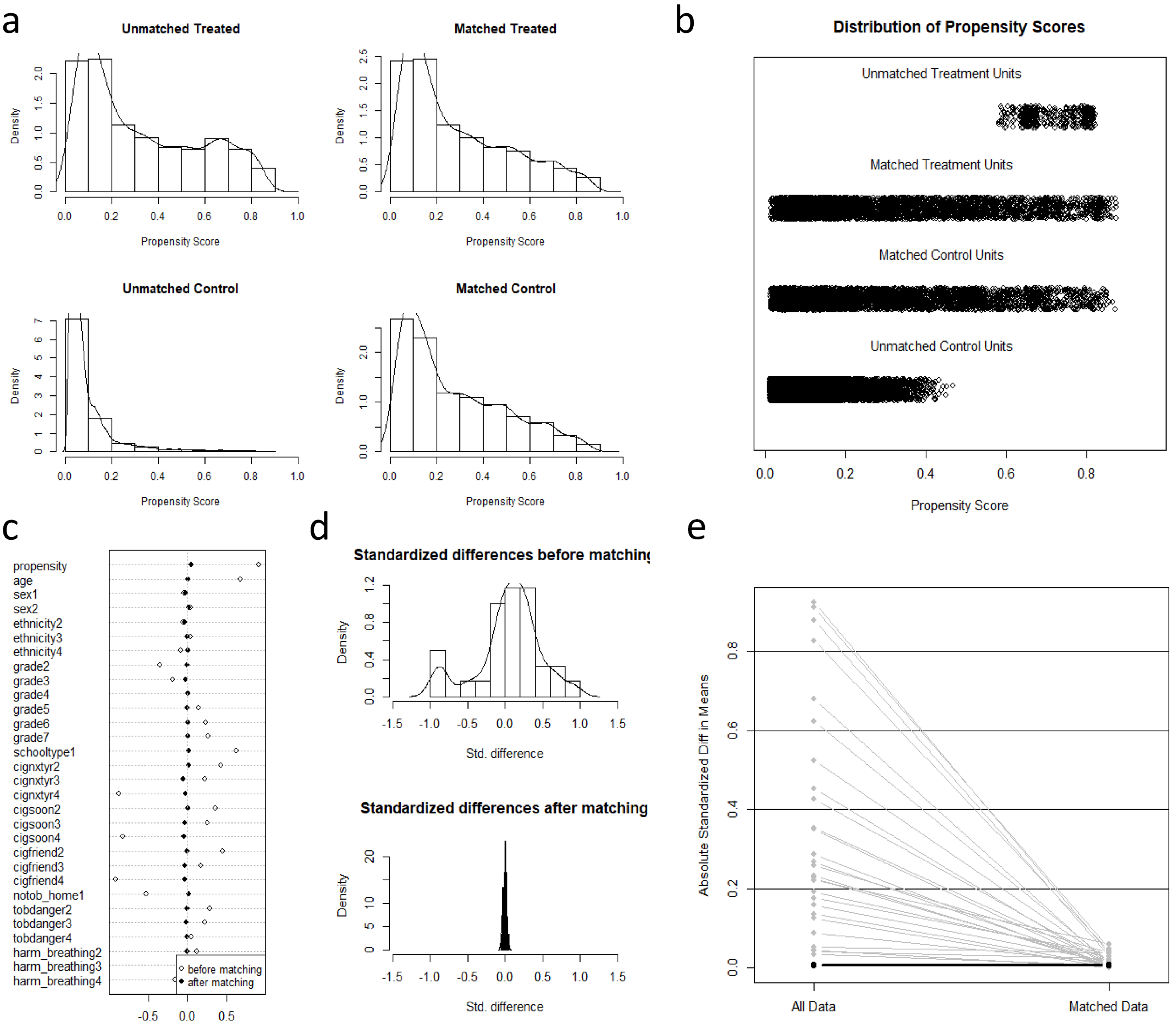

Figure S2 (a) Distribution of propensity scores of adolescents in 2014/2015 who used other combustible tobacco product first (treated) and those who did not (control) before and after matching overlaid kernel density estimate; (b) dotplot of adolescents in either matched or unmatched groups; (c) dotplot of standardized mean differences (Cohen's *d*) for all covariates before and after matching; (d) historgrams with overlaid kernel density estiamtes of standardardised differences before and after matching; (e) lineplot of standardardised differences before and after matching (bold: standardized differences that increase after matching)

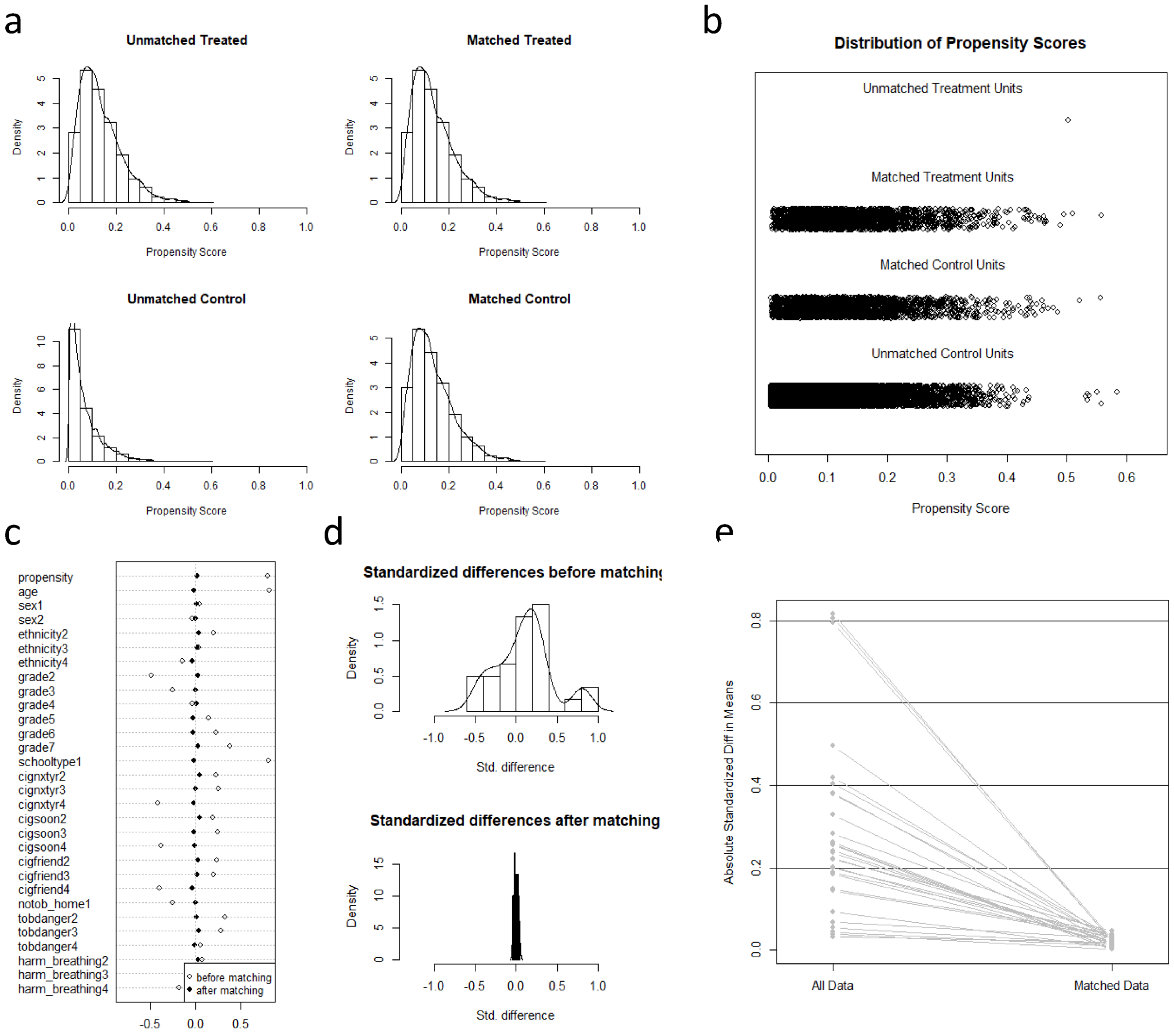

Figure S3 (a) Distribution of propensity scores of adolescents in 2014/2015 who used e-cigarette first (treated) and those who did not (control) before and after matching overlaid kernel density estimate; (b) dotplot of adolescents in either matched or unmatched groups; (c) dotplot of standardized mean differences (Cohen's *d*) for all covariates before and after matching; (d) historgrams with overlaid kernel density estiamtes of standardardised differences before and after matching; (e) lineplot of standardardised differences before and after matching (bold: standardized differences that increase after matching)

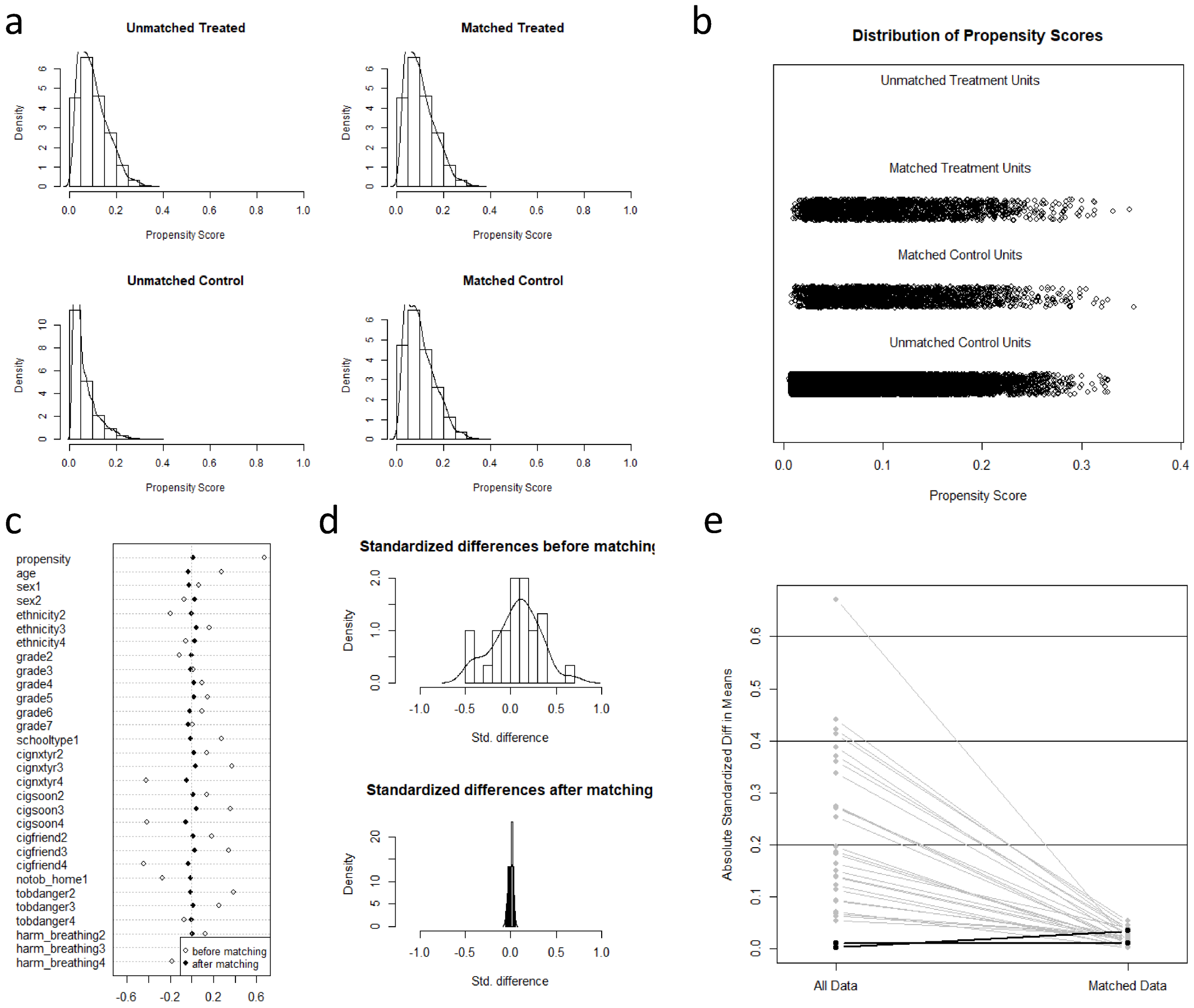

Figure S4 (a) Distribution of propensity scores of adolescents in 2014/2015 who used other non-combustible tobacco product first (treated) and those who did not (control) before and after matching overlaid kernel density estimate; (b) dotplot of adolescents in either matched or unmatched groups; (c) dotplot of standardized mean differences (Cohen's *d*) for all covariates before and after matching; (d) historgrams with overlaid kernel density estiamtes of standardardised differences before and after matching; (e) lineplot of standardardised differences before and after matching (bold: standardized differences that increase after matching)

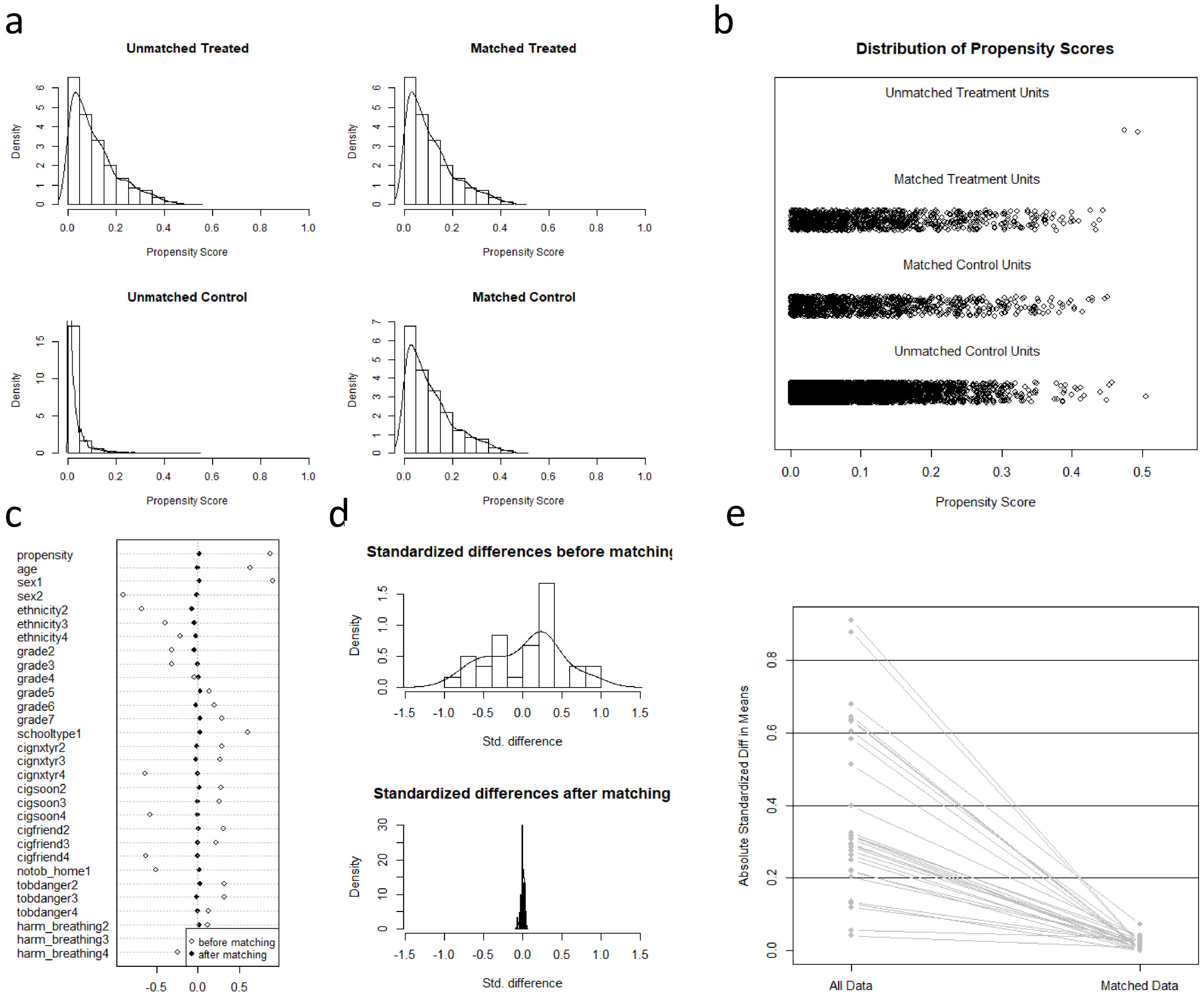

Figure S5: Prevalence of cigarette use by first product tried and propensity score matched controls selected from all four waves (2014-2017)

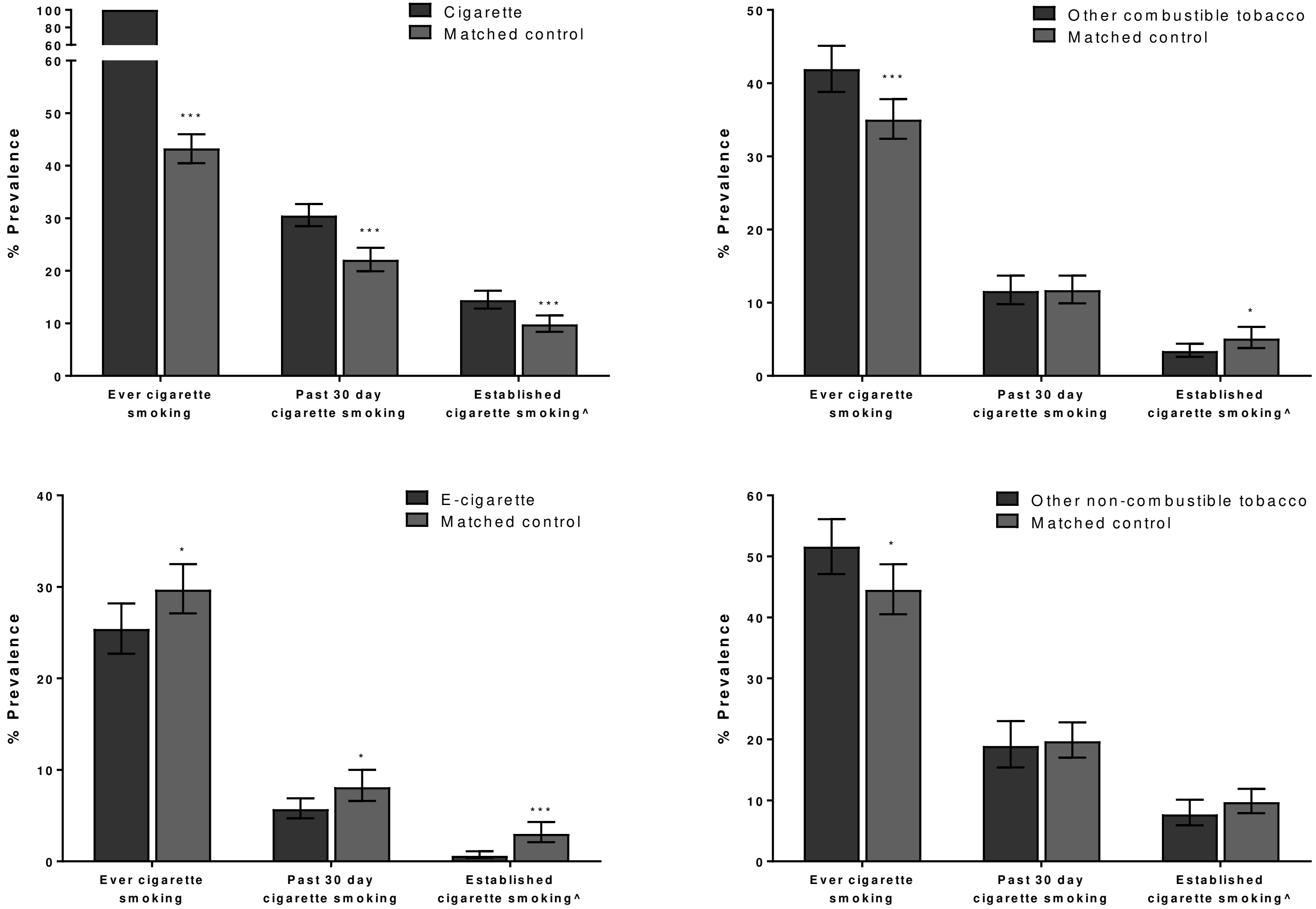

Error bars are 95%CI; ***P<0.001; *P<0.05; ^Smoking in past 30 days and at least 100 cigarettes in lifetime
